# Elevated Soybean Seed Oil Phenotype Associated with a Single Nucleotide Polymorphism in *GmNFR1α*

**DOI:** 10.3390/plants14233676

**Published:** 2025-12-03

**Authors:** Sri Veda Patibandla, Militza Carrero-Colón, Qijian Song, Quilin Qin, Elizabeth Clevinger, Hongyan Zhu, M. A. Saghai Maroof, Karen Hudson

**Affiliations:** 1 Department of Agronomy, Purdue University, West Lafayette, IN 47906, USA; patibans@purdue.edu; 2 Crop Production and Pest Control Research, USDA-ARS, West Lafayette, IN 47906, USA; 3 Soybean Genomics and Improvement Laboratory, USDA-ARS, Beltsville, MD 20705, USA; qijian.song@usda.gov; 4 Department of Plant and Soil Sciences, University of Kentucky, Lexington, KY 40546, USAhzhu4@uky.edu (H.Z.); 5 School of Plant and Environmental Sciences, Virginia Tech, Blacksburg, VA 24061, USA; ecleving@vt.edu (E.C.); smaroof@vt.edu (M.A.S.M.)

**Keywords:** mutagenesis, fine mapping, whole-genome resequencing, single nucleotide polymorphism, nodulation

## Abstract

Soybean seed composition, particularly the oil and protein content of the seed, has been a longstanding focus of research due to the economic and nutritional importance of these components for both feed and industrial applications. Through forward genetic screening of a mutagenized population derived from the soybean cultivar Williams-82, a mutant line designated PID 17238 was identified for high seed oil content. This phenotype is associated with a decrease in levels of protein with respect to Williams-82. The phenotype was mapped to chromosome 2 to a region near Satt459. Fine mapping and whole-genome resequencing were used to identify the causative mutation. Analysis of the resequencing data within the candidate region uncovered 55 sequence variants. *Glyma.02G270800* contained a single nucleotide polymorphism (SNP) within the coding sequence. *Glyma.02G270800* encodes a lysin motif (LysM) receptor-like kinase previously reported in the literature as *GmNFR1α*. Importantly, this locus is allelic to the well-characterized *rj1* locus, a recessive mutation known to cause a non-nodulating phenotype in soybean. Nodulation in soybeans, which enables nitrogen fixation, is crucial for protein synthesis in seeds, and the lack of nodulation may explain the lower protein content in PID 17238.

## 1. Introduction

With seeds that contain an average of 40% protein and 20% oil, soybean is a valuable crop. Understanding the function of the genes influencing soybean’s protein and oil content is an important step in creating new varieties with higher protein or oil content, which provides added value to consumers. Soybean seed composition is important because it directly impacts the nutritional value and industrial uses of soybeans. The diverse biochemical composition of soybean seeds makes them suitable for a wide range of industrial applications, including the production of plant-based meat alternatives and other processed food products [1,2].

Seed composition is a complex quantitative trait influenced by multiple genes and environmental conditions that complicate the selection process in breeding. The oil content of soybean ranges from 8.3 to 27.9%; the oil is generated and stored mainly as fatty acids (FAs), triacyclglycerols (TAGs), and tocopherols [3]. Soybean oil has many industrial applications, including biodiesel (a renewable fuel source), printing ink (an eco-friendly alternative to petroleum-based ink), industrial cleaners, and it is also an ingredient in cosmetic products. Increasing the seed oil content has been a major breeding objective for many years. Domesticated soybeans (*Glycine max*) have higher seed oil content than the wild soybean progenitor (*Glycine soja*). The *b1* allele, with the C to T mutation selected during domestication, appears to have pleiotropic effects, leading to increased oil content [4]. Recently, several additional genes have been reported to be associated with the increase in seed oil content. The *SWEET* sugar transporter genes, specifically *GmSWEET10a* and *GmSWEET10b,* have provided insight into how soybean seed oil levels are regulated. These genes are close homologues, expressed in the seed coat, and when overexpressed lead to a significant increase in oil content in seeds [5]. A study analyzing profitability of breeding soybean lines for high oil or high protein revealed that improving oil concentration has been economically less viable than increasing protein due to associated yield drag leading to lower profitability [6], indicating that new genetic solutions to this problem are still needed.

A significant variation exists in protein levels among wild and cultivated germplasm, offering a potential for breeding [7]. Protein content is negatively correlated with seed oil and yield, complicating breeding efforts. A study of soybean quantitative trait loci (QTL) identified a number of loci that demonstrated a consistent increase in protein concentration, but this trait was accompanied by reduced oil content, smaller seed size, lower yield, taller plants, and earlier maturity [8]. There are over 160 QTLs associated with seed protein content, with major consistent loci on chromosomes 15 and 20 [9]. The gene responsible for an important QTL for elevated protein on chromosome 20 was identified, which is a potential target for improving seed composition through marker-assisted selection or genome editing [10]. The challenges of improving seed composition include balancing the negative correlation between oil and protein, environmental effects on QTL expression of seed composition traits, the need to fine-map large QTL regions for practical breeding, and the genetic integration of mutant alleles into elite cultivars while maintaining agronomic performance [11]. The integration of genomics into soybean breeding has significantly advanced efforts to improve seed protein content by uncovering genes, QTLs, and molecular pathways. Marker-assisted selection (MAS), genomic selection (GS), and gene editing (CRISPR/Cas9) are crucial in order to improve traits in the future. Developing trait-specific QTLs and a deep understanding of gene function are key to balancing yield and nutritional quality.

Nodulation is the key trait that makes legumes important sources of protein and contributors to soil fertility. This process begins with the release of flavonoids that attract *Rhizobium*, triggering the expression of *nod* genes which encode Nod factors [12]. These are important for the establishment and the maintenance of the symbiotic relationship between rhizobia and legumes, which enables nitrogen fixation. Nod factors produced by rhizobia are recognized by LysM-domain receptor kinases such as NFR1/NFR5 [13]. Genetics and genomics have advanced the understanding of the pathways and genes involved in Symbiotic Nitrogen Fixation (SNF). The expression of the high-protein phenotype in soybean seed relies on nitrogen fixation in the nodules. Protein levels are significantly reduced in non-nodulating soybean lines, while hyper-nodulation of soybean has been considered in approaches to boost seed protein content [14]. These findings underscore the fact that high-protein soybeans depend on whole-plant nitrogen balance and physiology, and seed composition and development must be studied in this context.

Employing a genetic approach to improve protein and oil content, we conducted a mutant screen to obtain novel genetic variation with the potential to identify new alleles that could be utilized in the improvement of seed protein or oil content [15]. We identified one line (Plant ID# 17238) that demonstrated a recessive high-oil phenotype in seeds, with a concomitant decrease in protein levels. Here we describe the molecular identification of the gene responsible for the elevated oil phenotype isolated from this screen.

## 2. Results

### 2.1. A Locus Conferring Low Protein and High Oil Content Maps to Chromosome 2

In order to identify new genetic loci important for soybean seed protein and oil composition, we performed a forward genetic screen of a chemically mutagenized Williams-82 soybean population [16]. Among the mutants selected for seed composition traits, plant ID# (PID) 17238 was initially identified as having a higher oil content, which we observed after backcross to range from 5 to 22% higher than the Williams-82 wild-type over the growing seasons from 2020 to 2024, accompanied by a commensurate and statistically significant decrease in levels of seed protein (Table 1).

While Near-Infrared (NIR) spectroscopy was the rapid and non-destructive approach used for screening the population, the high-oil/low-protein phenotype was validated through proximate analysis using the Kjeldal method to ensure that any other changes in seed coat or seed reflectance unrelated to protein or oil composition were not responsible for the observed phenotype, and concurrence was observed for the chemical and NIR methods for protein and oil determination in this line (Table S1).

**Figure 1:**
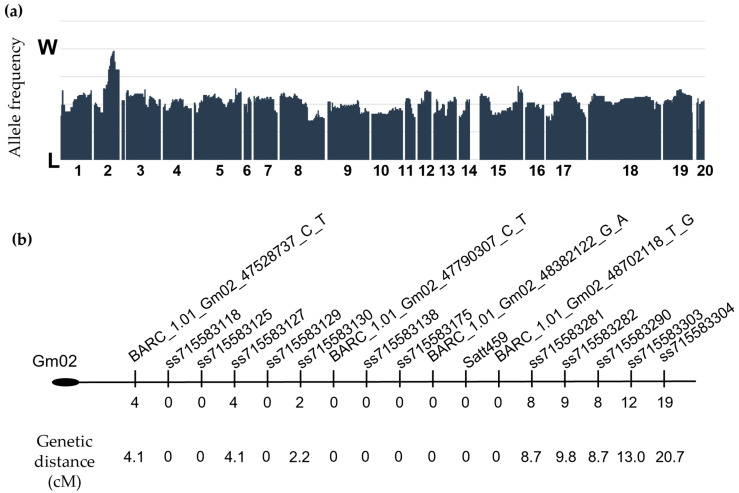
The high oil/low protein phenotype maps to chromosome 2. (**a**) Polymorphic markers (703) from the Illumina 3K BeadChip are plotted on the horizontal axis to indicate the genomic positions on 20 soybean chromosomes. Frequency of the parental alleles (Williams-82 = W, LG04-6000 = L) in the 17238 *×* LG04-6000 F_3_ mapping population (low protein individuals) is plotted on the vertical axis. (**b**) Fine mapping for positions on chromosome 2. BARC markers were obtained from the beadchip array data, while all the other SNPs were assayed with custom PCR-based markers designed to genome polymorphisms. Genetic distance is calculated in a population of 48 individuals.

In order to obtain a map position, the mutant was crossed to the well-characterized NAM parent line LG04-6000 [17,18]. Protein content in seeds from F_2_ plants demonstrated a bimodal distribution with a smaller peak at 33% (dry weight basis), suggesting a single recessive locus controls protein content in this line, while the oil content showed a continuous distribution (Figure S1). Fifty individual plants from the recessive class were selected for the mapping population, and a genotyping array was used to determine linkage of the trait to polymorphic SNP markers across the soybean genome (see Section 4). One strong peak for Williams-82 alleles in the recessive class was located on soybean chromosome 2 (linkage group D1b) around BARC_1.01_Gm02_48702118_T_G, with 49 of the 50 individuals found to carry homozygous Williams-82 alleles at this marker (Figure 1a). We checked the progeny from the one homozygous LG04-6000 individual and found that they did not show the low-protein or high-oil phenotype, so this line was assumed to be a contaminant. There were no other significant peaks of linkage or association with the Williams-82 genotype in the population.

Satt459 was the only microsatellite marker that was polymorphic between the Williams-82 and LG04-6000 parents in this region and confirmed linkage of Williams-82 alleles and the low-protein phenotype. To further narrow down the candidate region on Chr2, PCR-based SNP markers were designed based on polymorphisms from the 50K Illumina Bead Chip [18]. While we observed a few crossover events in this population with two of the markers (ss715583130 and ss715583127), we expect that this may be due to genomic re-arrangements as flanking markers were found to be homozygous for Williams-82 and a double crossover would not be expected at these distances (Figure 1b). With an effective population of 49 plants, we determined that the mutation responsible for the low-protein phenotype lay between bases 47,528,737 and 49,616,128 on chromosome 2 (Gmax1.01 genome version).

To determine the gene responsible for the low-protein/high-oil trait, we resequenced the whole genome of PID 17238 to identify polymorphisms in the interval of interest on chromosome 2. As the mutations in PID 17238 were created with *N*-nitroso-*N*-methylurea (NMU), we expected that induced mutations were likely to be single-base changes. We found 55 polymorphisms in the interval, but only two appeared to cause changes in amino acid sequence of predicted proteins (Table S2). PID 17238 carries a SNP (G to A) at position 51,674,794 on Chr 2 (*G. max* Wm82.a6.v1) within the gene *Glyma.02G270800*, resulting in an amino acid change of Glycine (G) to Glutamic acid (E) at amino acid residue 329. The *Glyma.02G270800* gene is annotated as chitin elicitor receptor kinase, expressed primarily in root tissues. The second polymorphism was a C to T transition at position 52,171,072 in *Glyma.02G276800* that caused a change from aspartic acid to asparagine at position 202 in the amino acid sequence. *Glyma.02G276800* is expressed in seeds and is part of a gene family that is well-conserved in legumes. While it has highly conserved domains near the substitution, position 202 is not universally conserved in the family (Figure S2). A SIFT (sorting intolerant from tolerant) analysis suggests that substitution of N for D at position 202 in the protein is likely to be tolerated [19]. No direct functional evidence of this gene family could be found, and the closest *Arabidopsis* ortholog shared an identity score of 49%.

### 2.2. Mutation in NFR1alpha

**Figure 2:**
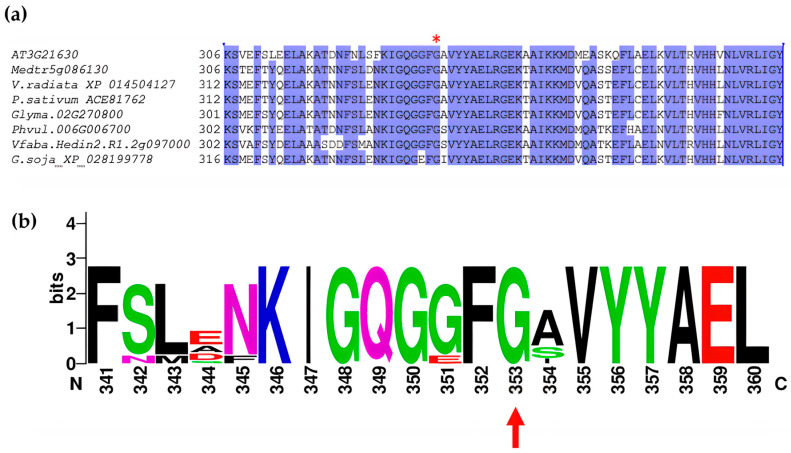
Polymorphism in Glyma.02g270800 associated with high oil phenotype. (**a**) Multiple sequence alignment of protein homologs was performed using the Clustal Omega web server 1.2.4. Protein sequences were obtained from Phytozome and included homologs from *Arabidopsis thaliana* (AT3G2160), *Phaseolus vulgaris* (Phvul.006G006700.1.p), *Vicia faba* (Vfaba.Hedin2.R1.1g259520.1), *Pisum sativum* (LOC127117980), Glycine soja (NFR1b), *Glycine max* (GLYMA.02G270800.1.P), *Vigna radiata* (LOC106764358), *Medicago truncata* (Medtr5g086130.1). Purple shading indicates identity >80%. Red asterix indicates the location of the substitution. (**b**) Weblogo indicating amino acid conservation. Red arrow indicates the conserved G mutated in plant ID 17238. The base numbering corresponds to the base in the multiple sequence alignment. Here the height of each letter in the sequence logo reflects the relative frequency and conservation of the corresponding amino acid at that position.

While the *Glyma.02G270800* gene is not expressed in seeds, it has been designated *GmNRF1α*, an LysM receptor-like kinase and is a part of the broader LysM-RLK gene family which comprises 27 members in soybean that are predicted to be involved in both immune and symbiotic signaling [20]. G329 is conserved across the gene family, particularly within subfamilies of LysM kinases [20] (Figure 2a,b). The *GmNFR1α* gene (*Glyma.02g270800*) is associated with the *rj1* locus controlling soybean nodulation, and contains a spontaneous single-base deletion that leads to early termination of the protein in germplasm line T201 [21]. Soybean *rj1* mutants have also been shown to have reduced yield as well as lower seed protein levels [22].

**Figure 3:**
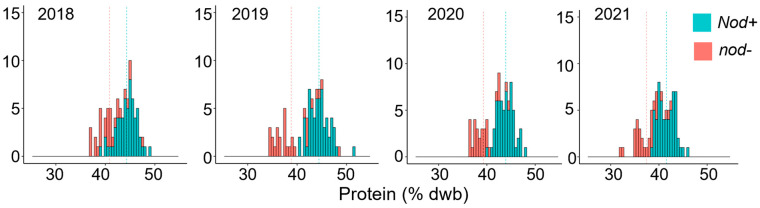
Co-segregation of protein content with nod- phenotype. Protein content over four seasons for nodulating (Nod+) and non-nodulating (nod-) RIL soybean lines from the cross PI507429 × PI399084. Dotted lines represent the mean for each phenotypic class. Protein phenotyping data is from [23], and the lines were tested for nodulation as described [25].

The region between markers BARCSOYSSR_02_1645 and BARCSOYSSR_02_1685 on chromosome 2, including the *rj1* gene, was recently identified in a QTL study as harboring a major locus controlling seed protein content in a cross between PI 507429 (Tousan 89, a low-protein line) and PI 399084 (a high-protein line), with LOD scores reaching as high as 17.48 and explaining up to 56.8% of the phenotypic variation for protein content [23]. The Satt459 SSR marker was identified within the maximum LOD QTL region and was also found to be linked with the low-protein/high-oil locus. In parallel, the Tousan 89 cultivar was found to lack root nodules in a study testing the specificity of the nodulation response [24]. These findings suggested that the low-protein phenotype in the RILs may be due to the failure to form root nodules as a consequence of mutation in *rj1*. We tested these recombinant inbred lines for nodule formation and determined that the low-protein/high-oil trait was found to be associated with the absence of nodules (Figure 3). This suggests that PI507429 (Tousan 89) may also carry an allele of *rj1* that impairs nodule formation and causes a reduction in seed protein levels. We resequenced the *Glyma.02g270800* gene in Tousan89 and found that it carries a polymorphism identical to the *rj1* reference allele (T201), a single base deletion resulting in a frame shift and early termination, indicating that *rj1* is likely the causative gene for the low-protein phenotype in this line.

**Figure 4:**
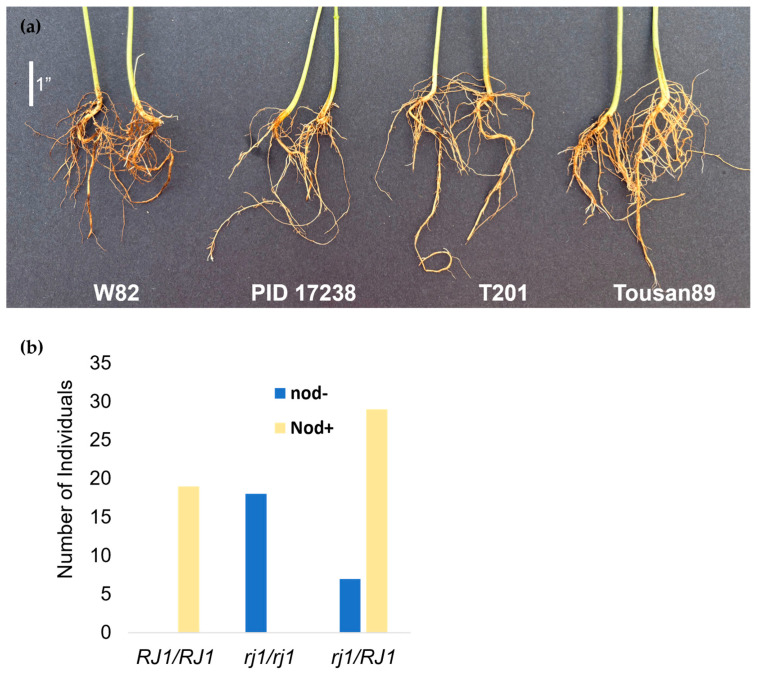
Nodules are absent in PID 17238. (**a**) 3 week-old plants from the Williams-82 wild type background, PID 17238, as well as the T201 and Tousan89 lines. (**b**) Absence of nodulation in a segregating population cosegregates with the *rj1* mutant gene.

To test whether the *rj1_G329E_* polymorphism affects nodulation in PID 17238, we examined roots of 3-week-old plants for nodulation. We observed that the PID 17238 mutants, like Tousan89 and the T201 accession, formed no nodules, while wild-type Williams-82 plants had an average of 10.3 readily visible nodules at this stage (Figure 4a). To test the association of this trait with the G329E polymorphism, we examined the progeny of a plant heterozygous for the *rj1_G329E_* mutant allele. We designed a co-dominant PCR marker to recognize the *rj1_G329E_* allele (Table S3). Of 74 F_3_, we observed 18 (24.3%) were mutant for the *rj1_G329E_*, 36 (48.6%) were heterozygous, and 19 (25.7%) were wild-type. None of the *rj1_G329E_* mutants formed nodules (Figure 4b) and all of the wild-type segregants formed nodules, supporting the linkage of the nodulation phenotype to the polymorphism in *rj1*. In this population, the wild-type plants had an average of 7.4 nodules, while the heterozygous plants formed fewer nodules, with an average of 4.8 at this stage. Seven heterozygous plants had no visible nodules (Figure 4b). We interpret this to indicate that one copy of the functional *GmNFR1α* gene is not sufficient for nodule formation at this stage of development. These results are consistent with *rj1_G329E_* being the polymorphism responsible for the loss of nodulation as well as low-protein seed phenotype in the PID 17238, which is supported by the function of soybean root nodules in nitrogen fixation and uptake as a requirement for high-seed protein.

## 3. Discussion

We identified a recessive low-protein trait mapped to a defined region on chromosome 2, in which we found only two polymorphisms that were predicted to affect protein-coding sequences. One of these polymorphisms occurred in the *Glyma.02G270800/GmNFR1α* gene, in a conserved domain. The absence of nodulation was further associated with protein content in an independent population. Aerial parts of PID 17238 were observed to be chlorotic at late stages of growth (Figure S3), which is a symptom of nitrogen deficiency due to absence of nodules. This phenotype aligns with physiological studies which investigate the effect of the absence of nodulation on photosynthetic response under elevated CO_2_ conditions [26]. Compared to the nodulating Williams-82 parent, a non-nodulating line showed a significant downregulation of photosynthesis under elevated CO_2_. This highlights the critical role of nodulation in maintaining sink strength and sustaining soybean productivity. Although a potential role for *Glyma.02G276800* in seed composition cannot be excluded without further genetic experiments such as introgression to separate the *Glyma.02G276800* and the *rj1* polymorphisms, taken together, our results suggest that it is likely that the *rj1* gene is the most compelling candidate to explain the phenotypes that we observe in PID 17238.

The *rj1* gene is highly conserved in soybean accessions, underscoring the importance of nodule formation to soybean growth and development. An examination of the soybean allele catalog (https://soykb.org/SoybeanAlleleCatalogTool/, accessed on 12 February 2025) shows 17 potential polymorphisms in the *rj1* gene identified in sequenced soybean accessions (Table S4) [27]. None of these are allelic to the polymorphisms in PID 17238, consistent with *rj1_G329E_* being a novel mutation induced by the mutagen. The only previously described polymorphism affecting nodulation within the sequenced accessions is the polymorphism found in T201 (PI 548193).

Understanding the role of the *Rj* genes can help to improve nitrogen fixation efficiency by preventing nodulation with ineffective rhizobial strains. There are three recessive *rj* genes (*rj1*, *rj5*, and *rj6*) which lead to non-nodulation, due to mutation in genes encoding Nod factor receptors which are essential for recognizing rhizobial signaling molecules [28]. Dominant *Rj* genes (*Rj2*, *Rj3*, *Rj4*, and *Rfg1*) restrict nodulation with specific *Bradyrhizobium* or *Sinorhizobium* strains. Apart from these, *rj7* is a hypernodulation mutant, a recessive allele that affects the autoregulation of nodulation (AON) system. The T201 *rj1* mutant (PI548193) has a single-base-pair deletion in the fourth exon of *Glyma.02g270800* resulting in a frameshift and a non-functional protein lacking the C-terminal kinase domain, leading to a non-nodulating phenotype with almost all *Bradyrhizobium* and *Sinorhizobium* strains [21]. Another accession (T3791) carries an identical nucleotide deletion in the fourth exon of Glyma.02g270800 [29]. Another known mutation, *nod49,* involves the deletion of a T in exon 5 of the *GmNFR1α* coding sequence, causing a frameshift and resulting in premature protein termination [14]. The amino acid change in PID 17238 occurs in exon 5 within a conserved domain identified as the G-Rich region, proposed to be required for kinase function [20,30] and is the only known missense allele of *rj1*. The *GmNFR1β* gene (*Glyma.14g046200*), located on chromosome 14, while highly similar to *GmNRF1α* with 89% amino acid identity, is expressed at significantly lower levels, and some cultivars carry natural missense alleles in *GmNFR1β* which do not appear to affect nodulation in the presence of functional *GmNRF1α* [14]. Thus, *GmNRF1α* is considered the primary contributor to Nod factor perception [28]. Over-expression of *GmNRF1α* in soybean has been shown to result in a significant increase in number of nodules and plant nitrogen content [14].

This finding emphasizes how variations in nodulation genes can be manipulated to balance protein and oil accumulation from a breeding perspective. The functional *GmNFR1α* allele might be backcrossed into low-protein, high-oil germplasm to investigate nitrogen fixation restoration and its effect on yield and protein synthesis. Additionally, these introgression materials would provide comprehensive field assessments under different nitrogen regimes to ascertain the physiological boundaries of carbon–nitrogen partitioning between protein accumulation and oil [22,26]. To improve downstream signaling and LysM receptor function, future research could use CRISPR/Cas9-mediated editing techniques and genomic selection. This could result in partial or conditional nodulation phenotypes that maintain sufficient nitrogen fixation while encouraging carbon flow toward lipid biosynthesis [11,14,28]. By connecting molecular insights with useful breeding results, these tactics would support soybean improvement initiatives that attempt to strike a balance between productivity and seed composition quality.

## 4. Methods and Materials

### 4.1. Plant Materials and Growth Conditions

A population of Williams-82 was mutagenized with NMU. The progeny of these mutants were planted in 1.8 m row plots at the Agronomy Center for Research and Education field in West Lafayette, Indiana. For screening of seed protein and oil levels, seeds were measured in 15 seed bulks on the Perten DA 7250 NIR analyzer (Perten, Springfield, IL, USA) [15]. Seed chemical analysis for crude protein (Kjeldahl method) was provided by the University of Missouri Chemical Analysis Facility (Columbia, MO, USA) (https://aescl.missouri.edu/Prox.html, accessed on 12 February 2025). We performed one backcross using Williams-82 as a female parent to reduce effects from potential multiple mutations in the genome and selected low-protein/high-oil progeny from this cross for propagation and further study. To examine the linkage of the nodulation phenotype with the allele in the 17238 mutant, we identified an individual heterozygous for *rj1_G329E_* from an F_2_ population from a cross (PID 17238 ×Prize) and allowed it to self-pollinate in the greenhouse to obtain fresh seed for nodulation assays. These F_3_ plants were grown in 12 hL/12 H dark cycles at 25 °C and 75% relative humidity in growth chambers in a non-sterile soil mixture containing 1/3 sand, 1/3 topsoil, 1/3 potting soil (BM7, Berger.ca, Quebec, Canada) mix (no inoculum was included). Lighting was provided by a combination of incandescent and fluorescent bulbs at an intensity of 350 μmol m^−2^ s^−1^. After 3 weeks, plants were uprooted and washed to visualize nodule growth. For the PI507429 x PI399084 RIL population, nodulation assays were performed on F_12_ generation plants in vermiculite with inoculation using methods described previously [25].

### 4.2. Genetic Mapping, Sequencing, and Genotyping

To identify the gene responsible for the high-oil phenotype, a mapping population was developed by crossing PID 17238 with LG04-6000 [17]. F_2_ from this outcross population were planted in the field in 2020, and seed protein and oil were measured in F_3_ seed samples from each individual. A total of 279 F_2_ plants from two different F_1_ plants were phenotyped and demonstrated the same segregation pattern (which we interpreted to mean that they could carry the same allele combinations at the loci responsible for the trait). A clear bimodal distribution for seed protein content was observed in the F_3_ bulks (Figure S1). DNA was extracted from a total of 55 samples from the recessive peak that were selected for low protein content (protein < 36.8%) using Qiagen DNeasy Plant kit (Qiagen, Germantown, MD, USA) using the standard kit protocols, and the Illumina Infinium HTS iSelect custom array for soybean (3K) (Illumina Inc., San Diego, CA, USA) was used to detect linked markers for determination of an approximate map position. Only ~700 markers polymorphic between the parents (Williams-82 and LG04-6000) were found to be informative.

For fine linkage mapping, we designed SNP markers (primer sequences used for sequencing and genetic mapping are listed in Table S3). SNPs inferred to be polymorphic between LG04-6000 and Williams-82 were initially selected from the SoySNP50K dataset available on SoyBase (soybase.org), and corresponding dCAPS markers [31] to distinguish these alleles were designed for genotyping using the online primer design tool, Primer 3 (https://bioinfo.ut.ee/primer3-0.4.0/, accessed on 12 February 2025). The primers were tested with a temperature gradient (52–54–56–58–60) to identify the optimum annealing temperature. SNPs polymorphic between Williams-82 and LG04-6000 were further tested across all the individuals in our segregating population. The PCR conditions for the gradient are as follows: initial denaturation for 1 min at 95 °C, then 34 cycles of 15 s at 95 °C, 15 s at a gradient temperature of 52–54–56–58–60 °C for annealing followed by 30 s at 72 °C. The final extension was 7 min at 72 °C. After these steps, the samples were held at 4 °C indefinitely. A PCR-based marker to distinguish the *rj1_G329E_* polymorphism was designed using the same marker design protocol is also described in Table S3.

For genome resequencing, plant DNA was prepared using the CTAB method [32] and short read sequencing was provided by a commercial service. 154 million paired-end reads (150 bp) were obtained, with 93.3% > Q30, providing an estimated coverage of 40-fold of the soybean genome. Reads were mapped to Williams-82 genome version 6, and SNPs were called using the Sentieon (version 202308.03) DNAseq pipeline according to practices described elsewhere [33].

## 5. Conclusions

In the chemically mutagenized soybean line PID 17238, we identified a novel allele of GmNFR1α (*Glyma.02G270800*) that is responsible for a high-oil/low-protein phenotype in seeds. Within a conserved domain of the LysM receptor-like kinase, a single nucleotide polymorphism that results in an amino acid substitution was identified by fine mapping and whole-genome resequencing. Because this mutation also interferes with soybean nodulation, we infer that both nitrogen fixation and seed protein accumulation are decreased in the mutant. These results demonstrate how nodulation, nitrogen balance, and seed composition are interrelated, highlighting the significance of whole-plant physiology in breeding for better industrial and nutritional qualities. In addition to providing a distinct genetic cause for the phenotype that we observed, this study deepens our knowledge of the trade-offs between soybean oil and protein and offers a useful resource for upcoming attempts to modify seed composition via gene editing or genomics-assisted breeding techniques.

## Figures and Tables

**Table 1:** Average and standard deviation for protein and oil levels (dry weight basis) for bulk seed samples from individual plants of the mutant line over three seasons, compared to Williams-82. Statistical significance computed by *t*-test ** *p* < 0.005, * *p* < 0.05.

	Williams-82		Williams-82 × 17238	
Year	Protein	Oil	Protein	Oil
2020	41.61 ± 2.1	18.92 ± 0.9	32.91 ± 1.1 **	20.54 ± 0.5 **
2021	41.97 ± 2.4	21.03 ± 1	35.5 ± 2.9 *	22.21 ± 0.4
2022	43.1 ± 1.6	20.3 ± 0.8	31.2 ± 2.0 **	23.1 ± 0.84 **
2023	38.8 ± 1.4	21.6 ± 0.7	30.5 ± 2.0 **	25.0 ± 0.9 **
2024	38.09 ± 1.3	23.9 ± 0.5	29.6 ± 0.9 **	24.8 ± 0.7 *

## Data Availability

Raw data supporting the conclusions of the article will be made available upon request.

## Supplementary Materials

The following supporting information can be downloaded at https://www.mdpi.com/article/10.3390/plants14233676/s1, Table S1: Proximate seed composition analysis in PID 17238; Table S2: Candidate SNPs in the defined interval on chromosome 2 (Excel spreadsheet); Table S3: Primer sequences and genotyping assays; Table S4. SNPs in *Glyma.02G270800* in the Soybean Allele Catalog (Excel spreadsheet); Figure S1: Monogenic segregation of protein content in the F_2_ mapping population; Figure S2: Multiple sequence alignment for *Glyma.02G276800*; Figure S3. Chlorosis in aerial parts of PID 17238.
