# Supplementary material for: Elevated Soybean Seed Oil Phenotype Associated with a Single Nucleotide Polymorphism in GmNFR1α
Source: Plants (Basel). 2025 Dec 3;14(23):3676. doi: 10.3390/plants14233676 (PMC12694145; doi:10.3390/plants14233676)
Supplement: Supplementary file 1 [file plants-14-03676-s001.zip › Figure S1.pdf]

(a)

(b)

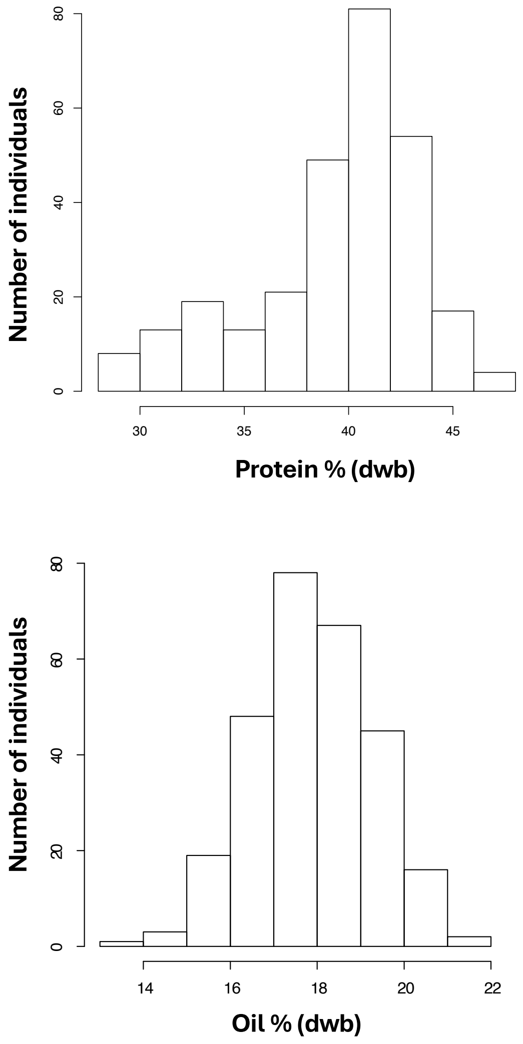

Figure S1: Monogenic segregation of protein content in the F_2 mapping population. Low protein phenotype segregates as a single, recessive trait. **(a)** The distribution of protein content across the F_3 seed bulk samples (derived from the cross 17238 x LG04-6000). Total protein ranges between 28% - 47%, evidence of bimodal distribution with a primary peak at 40% and a recessive peak at 33-35%. For mapping plants with < 36.8% protein were selected. **(b)** The distribution of oil content in the F_3 samples ranging from approximately 14%-22%.
