# Supplementary material for: Elevated Soybean Seed Oil Phenotype Associated with a Single Nucleotide Polymorphism in GmNFR1α
Source: Plants (Basel). 2025 Dec 3;14(23):3676. doi: 10.3390/plants14233676 (PMC12694145; doi:10.3390/plants14233676)
Supplement: Supplementary file 1 [file plants-14-03676-s001.zip › Figure S2.pdf]

```
                                                        ↓ D202N
Lalb_Chr04g0259481          MGFSQELATVALILNEGRVEKSVAWLFEGGEESDIHKDKDVGGGNLKIDISEELARIADL    229
Vfaba.Hedin2.R1.1g264240.1  MGFSQERATMALVLNEGRVEESVAWLFESGEEDGQNDKTAGRGGNLKIDISEELARIADM    229
Glyma.02G276800.1.p         MGFSHERATMALILNEGRVEESVAWLFEGGEDADGNKDTNIGRGNLKIDISDELARIADM    228
Vigun08g180800.1.p          MGFSHERATMALILNEGRVEESVAWLFEGGEEADGNKDTNIRRGNLKIDISDELAQIADM    229
Pl08G0000341800.1.v1        MGFSHERATMALILNEGRVEESVAWLFEGGEEADGNKDTNIYRGNLKIDISDELVQITDM    239
Phvul.008G204600.1.p        MGFSHERATMALILNEGRVEESVAWLFEGGEEADGNKDTNIYRSNLKIDISDELVQITDM    229
Phacu.CVR.008G263500.1      MGFSHERATMALILNEGRVEESVAWLFEGGEEADGNKDTNIYRGNLKIDISDELVQITDM    229
                            ****:* **:**:*******:*******.**: . :...    .*******:**.:*:*:
```

Figure S2. Multiple sequence alignment of protein homologs of Glyma.02G276800 was performed using the Clustal Omega web server. Protein sequences were obtained from Phytozome and included homologs from *Lupinus albus* (Lalb_Chr04g0259481), *Vicia faba* (Vfaba.Hedin2.R1.1g264240.1), *Glycine max* (Glyma.02G276800.1.p), *Vigna unguiculata* (Vigun08g180800.1.p), *Phaseolus lunatus* (Pl08G0000341800.1.v1), *Phaseolus vulgaris* (Phvul.008G204600.1.p), *Phaseolus acutifolius* (Phacu.CVR.008G263500.1).
