# Supplementary material for: Elevated Soybean Seed Oil Phenotype Associated with a Single Nucleotide Polymorphism in GmNFR1α
Source: Plants (Basel). 2025 Dec 3;14(23):3676. doi: 10.3390/plants14233676 (PMC12694145; doi:10.3390/plants14233676)
Supplement: Supplementary file 1 [file plants-14-03676-s001.zip › Figure S3.pdf]

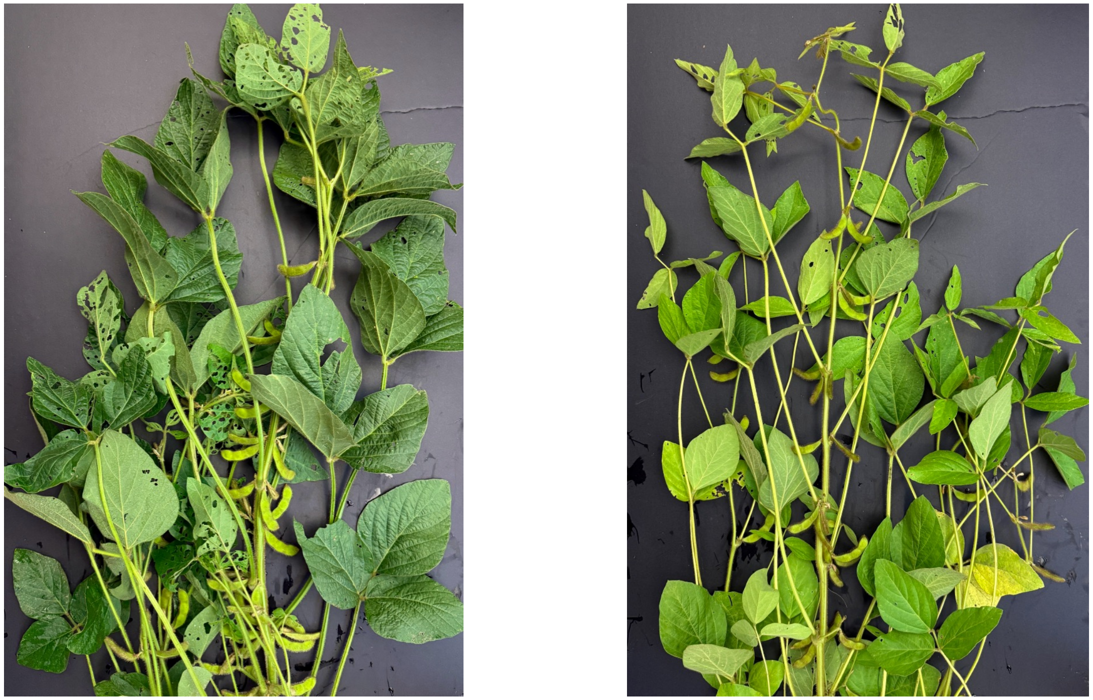

Williams 82 **PID 17238**

Figure S3: Aerial parts of Williams-82 and PID 17238 at R6 growth stage. Mutant shows chlorosis symptoms.
