# Supplementary material for: Elevated Soybean Seed Oil Phenotype Associated with a Single Nucleotide Polymorphism in GmNFR1α
Source: Plants (Basel). 2025 Dec 3;14(23):3676. doi: 10.3390/plants14233676 (PMC12694145; doi:10.3390/plants14233676)
Supplement: Supplementary file 1 [file plants-14-03676-s001.zip › Table S1.pdf]

		NIR Spectroscopy		Chemical Analysis	
Genotype		Protein(%DWb)	Oil(%DWb)	Protein(W/W%)	Oil(W/W%)
Williams 82	W-82	42.68	21.31	40.95	20.86
17238	W-82 X 17238.5	29.34	23.62	29.17	22.27

Supplemental Table S1: Correspondence of NIR and proximate analysis in Williams-82 and the 17238 mutant line. %DWb – Dry weight basis (percentage of substance calculated after removing all the moisture from the sample), %W/W – Weight/weight percentage (Represents the amount of substance in relation to the total mass of the sample including moisture or solvents).
