# Supplementary material for: Elevated Soybean Seed Oil Phenotype Associated with a Single Nucleotide Polymorphism in GmNFR1α
Source: Plants (Basel). 2025 Dec 3;14(23):3676. doi: 10.3390/plants14233676 (PMC12694145; doi:10.3390/plants14233676)
Supplement: Supplementary file 1 [file plants-14-03676-s001.zip › Table S3.pdf]

SNP	Forward Primer	Reverse Primer	Restriction enzyme
ss715583118	TGCCAACTCCGACATTGTTA	TGAATGTCCGCGCGTGCGAAATCTG	DdeI
ss715583125	ATCAATTTGTGCCCATCCAT	CCAAGGTACATATCCCATCTATTCCTA	AvrII
ss715583127	GCAAATGAAATGGCGGCGTA	GGCAGCGTAACAAGCAATGT	DdeI
ss715583129	CCCTCCTTCGTAAATTTGCCTTAT	GCTCTCAGTCACTTGTGTCAATG	Psi I
ss715583130	ggacatccagcccaattaga	AGGCCCATTGGGAGGTTGGT	Bsm AI
ss715583138	AACTCAGTCTCACGCCATGCTAG	AATCTTTCGAGATACCGAGATTT	NheI
ss715583281	GAAACCGGAACCGCCGCGGC	GGTTCAAGCCGATTTGGTT	NotI
ss715583175	ATAGAAGAACACAGCACATATGTTAA	ACGAACCCGAAAAGAAGAGG	HpaI
ss715583281	GAAACCGGAACCGCCGCGGC	GGTTCAAGCCGATTTGGTT	NotI
ss715583282	TTCTTATGATATCTTTGTACCATG	tttgatttggtttgatttTTAGCA	Nco I
ss715583290	AACAGATGGGGACCTCTCCT	GTTGACTCGGTTCCCCTCCTGC	HhaI
ss715583303	TGAGGTTTTCACCTGTGACG	ACGACTTGTACGTCTCCATCTCGGGGT	Hph I
ss715583304	GTACCAATCACTATCAACCTTTTTGGTAAC	GGAGGTGGTGGTACCAGAGA	BstEII
Rj1$_{G329E}$ genotyping	TCAGTTCTGCATAATAGAaAGCT	TAGCCAAGGCTACAAATAACTTT	HindIII

***Table S3*. Primer sequences (5' -3') used to fine map and genotype *rj1*.**
